# Generalizability of YOLOv11 models for mesiodens detection in pediatric panoramic radiographs

**DOI:** 10.1186/s12903-026-07713-z

**Published:** 2026-01-28

**Authors:** Henri Hartman, Adinara Savero, Tjinta Kaulika Tamim, Farina Pramanik, Saiful Akbar, Denny Nurdin, Arlette Suzy Setiawan

**Affiliations:** 1 Doctoral Program, Faculty of Dentistry, Universitas Padjadjaran, Bandung, Indonesia; 2 Department of Pediatric Dentistry, Faculty of Dentistry, Universitas Jenderal Achmad Yani, Cimahi, Indonesia; 3 Faculty of Dentistry, Universitas Jenderal Achmad Yani, Cimahi, Indonesia; 4 Department of Dentomaxillofacial Radiology, Faculty of Dentistry, Universitas Padjadjaran, Bandung, Indonesia; 5 School of Electrical Engineering and Informatics, Institut Teknologi Bandung, Bandung, Indonesia; 6 Department of Conservative Dentistry, Faculty of Dentistry, Universitas Padjadjaran, Bandung, Indonesia; 7 Department of Pediatric Dentistry, Faculty of Dentistry, Universitas Padjadjaran, Bandung, Indonesia

**Keywords:** Mesiodens, Pediatric dentistry, Panoramic radiography, Deep learning, YOLO

## Abstract

**Background:**

Mesiodens is a type of supernumerary tooth in the anterior maxilla with various prevalences. To prevent complications in the future, accurate and precise detection is needed.

**Objective:**

This study aimed to evaluate and compare YOLOv11-based convolutional neural network (CNN) models for mesiodens detection in pediatric panoramic radiographs using two cloud-based platforms, Roboflow and Ultralytics.

**Design:**

This study involved 480 pediatric panoramic radiographs, consisting of 240 mesiodens and 240 no mesiodens images, annotated using Roboflow, with a region of interest (ROI) focused on the anterior maxillary area. The dataset was divided into training (70%), validation (20%), and testing (10%) subsets. Model performance was evaluated using mean average precision (mAP), precision, recall, and F1-score.

**Results:**

The YOLOv11 Accurate model trained on the Roboflow platform achieved the highest validation mAP50 at 99.2% and recall at 100%. However, its performance declined on inference data, where the F1-score was 84.30%. In contrast, the YOLOv11l model trained on the Ultralytics platform showed more stable performance: its validation mAP was 99.3%, precision was 99.11%, and recall was 94.57%, while the inference F1-score was 96.78%, showing robust generalizability and supporting its suitability for clinical practice.

**Conclusion:**

YOLOv11l demonstrated the most reliable balance between validation and inference performance, suggesting suitability for clinical application. These results highlight the importance of model generalization rather than peak validation metrics. Future studies should therefore evaluate multicenter datasets and broader clinical settings to confirm robustness and applicability in diverse pediatric populations.

## Introduction

 Supernumerary teeth (SNTs) have been known for centuries, yet early detection continues to pose challenges for clinicians [1]. These extra teeth may occur in the maxilla or mandible and often emerge alongside or beyond the normal dentition. Mesiodens is the most prevalent among various types, with reported incidences ranging from 0.15% to 3.9%[2–6]. The etiology of mesiodens remains a topic of debate, with several theories proposed, including atavism, dichotomy, and hyperactivity of the dental lamina [7, 8]. Systemic conditions such as cleidocranial dysplasia, Gardner syndrome, cleft lip, and palate are also associated with an increased risk of supernumerary teeth, including mesiodens [5, 8]. A genetic predisposition is also evident, with patterns observed among siblings, twins, and sex-linked inheritance [9]. 

Currently, Cone-Beam Computed Tomography (CBCT) is considered the gold standard for accurately detecting the location of mesiodens in children. Nevertheless, panoramic radiographs remain the diagnostic tool of choice in many settings due to their accessibility, simplicity, and lower cost [10]. Prompt identification of mesiodens is essential to prevent potential complications, including delayed eruption of adjacent teeth, diastema, impaction of adjacent permanent teeth, root resorption, and the formation of cystic lesions. Precise identification is fundamental to enabling the administration of appropriate and effective treatment strategies[7, 11–13].

Recent advancements in artificial intelligence (AI), particularly in deep learning, have shown promise in aiding the detection of mesiodens [14]. Deep learning models are capable of automatically extracting complex features from image data [15, 16]. Recent studies have shown that deep learning models can reliably assist in identifying dental anomalies. Within this field, You Only Look Once (YOLO)-based convolutional neural networks (CNNs) have been applied to mesiodens detection with encouraging outcomes. For example, Kuwada et al. (2020) demonstrated that DetecNet and AlexNet could accurately recognize supernumerary teeth on panoramic radiographs, while Ha et al. (2021) reported high diagnostic accuracy for mesiodens classification using a YOLOv3 framework [1, 17]. More recently, Dai et al. (2023) confirmed that YOLO could even localize mesiodens automatically with strong precision [18]. These findings collectively underline YOLO’s potential as a valuable tool in dental radiology [19]. 

Since then, the YOLO architecture has evolved, and the latest version, YOLOv11, introduces improvements in network structure, including enhanced backbone and detection head design, as well as faster training and inference speed [1, 16–18]. Although not yet widely applied to mesiodens detection, its capabilities indicate strong potential. In addition to YOLO, other CNN architectures such as SqueezeNet, ResNet, AlexNet, and InceptionV3 have been applied to dental anomaly detection, demonstrating that deep learning can adapt well across different models and imaging scenarios in dentistry [1, 15, 20–22]. 

In this study, our primary objective was to optimize mesiodens detection using state-of-the-art YOLOv11 architectures. To achieve this, we implemented and compared models trained on two widely used cloud-based deep learning platforms, Roboflow and Ultralytics. This comparison was intended not as an end in itself, but to evaluate whether platform-specific differences in data handling and training pipelines influence the diagnostic performance of YOLOv11 for mesiodens detection. Our approach focuses on optimizing detection performance through advanced CNN architecture, aiming to provide a practical and scalable solution for clinical use. To the best of our knowledge, this is the first comparative evaluation of YOLOv11 architectures in pediatric mesiodens detection using both Roboflow and Ultralytics platforms.

## Materials and methods

The present study was designed as a retrospective observational analysis using pediatric panoramic radiographs. The primary objective was to evaluate the performance of YOLOv11-based convolutional neural network (CNN) models in detecting mesiodens, extending our previous research on its prevalence toward the development of automated diagnostic tools in pediatric dentistry.

The radiographs were retrospectively collected from four university-affiliated dental hospitals in West Java, Indonesia, representing diverse clinical environments and routine pediatric imaging workflows. All images were acquired using digital panoramic radiography systems, with complete device information available for each site: Instrumentarium OP300 Maxio Panorex (RSGM Universitas Padjadjaran), SOREDEX Cranex 3D PAN (RSGM Universitas Jenderal Achmad Yani), VATECH 3D (RSKGM Bandung), and Morita XH-550 (RSGM Maranatha). Detailed exposure settings such as kVp, mA, and exposure time were only partially accessible due to retrospective archival limitations, so these parameters were not included as analytical variables. The availability of machine-type metadata provides contextual insight into the diversity of imaging systems used across institutions while maintaining clarity regarding the constraints of the dataset.

The methodology followed a structured workflow that included ethical approval, dataset preparation and annotation, region of interest (ROI) definition, model development on two cloud-based platforms, and evaluation using standard object detection metrics. Each of these stages is described in detail in the following subsections.

### Ethics

This was a retrospective study using pediatric panoramic radiographs to develop and evaluate CNN models for mesiodens detection. Ethical approval was granted by the Health Research Ethics Committee of Universitas Padjadjaran (Approval No.1245/UN6.KEP/EC/2023). The study was conducted in accordance with the principles of the Declaration of Helsinki. As the study was retrospective, no additional informed consent was required.

### Data annotation and preparation

The dataset was derived from our previous cross-sectional prevalence study involving children aged 6–12 years. From the total of 1,184 pediatric panoramic radiographs evaluated in that study, mesiodens was identified in 78 patients (6.6%), with a higher prevalence in males (9.4%) and the highest frequency observed among 7-year-old children. Among these patients, 59 presented with a single mesiodens, whereas 19 exhibited multiple mesiodens. As 78 positive cases were insufficient for model training, limited augmentation was applied to preserve anatomical fidelity while increasing the number of positive samples to 240. The ‘No Mesiodens’ category was then randomly sampled to match this number, resulting in a balanced dataset of 240 versus 240 images for model development. The train–validation–test split was generated automatically by the Ultralytics and Roboflow workflows using predefined ratios.

A qualitative image quality assessment was performed prior to annotation. Radiographs were excluded if they showed severe motion blur, prominent artefacts, truncated or unclear anterior maxillary regions, extreme under- or overexposure, or anatomical conditions that prevented reliable mesiodens assessment (such as cleft lip or missing maxillary anterior teeth). Only images meeting a minimum standard of diagnostic acceptability were included. Following this screening, two pediatric dentists and one dentomaxillofacial radiologist with more than five years of clinical experience annotated the images using Roboflow, following the COCO segmentation format, a widely adopted standard structure for object detection datasets.

The dataset was systematically divided into training (70%), validation (20%), and testing (10%) subsets. To ensure transparent data handling and avoid optimization bias, the dataset split was performed once using a fixed random seed before model development. The inference subset remained completely unseen during training and validation and was used only once for final evaluation. No hyperparameter tuning, model selection, or confidence threshold adjustment was performed using the inference/test data. External validation from independent institutions was not available for this study. Roboflow performed randomization without extensive editing or enhancement. Augmentation strategies included horizontal flipping, brightness adjustments between − 15% and + 15%, and exposure shifts between − 10% and + 10%, without resizing or cropping the anatomical region. A total of 240 images depicting mesiodens were utilized in this study.

### Region of interest setup

To improve training efficiency, a region of interest (ROI) was defined in the anterior maxillary area, where mesiodens are most frequently found. Regions of interest were defined entirely through annotations rather than through image cropping. Two object classes were labeled: (1) ‘Mesiodens,’ annotated using polygonal contours to delineate the supernumerary tooth morphology precisely, and (2) ‘No Mesiodens,’ annotated using a standardized bounding box extending from the distal–apical border of the right maxillary canine to the distal–occlusal border of the left maxillary canine. This bounding box restricted the detection focus to the maxillary anterior region, preventing false detections in non-relevant areas while preserving whole-image context. All annotations were reviewed and finalised in expert consensus using a uniform anatomical rule, making a formal inter-rater reliability calculation unnecessary.

**Fig. 1:**
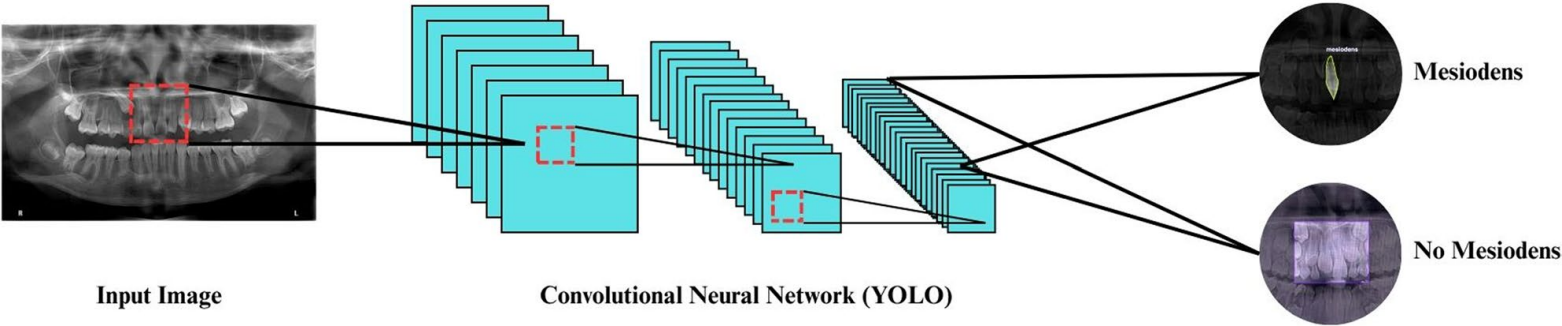
Workflow of the YOLO-based convolutional neural network for mesiodens detection

The ROI extended horizontally across the anterior maxilla and vertically was limited to the maxillary region, thereby excluding irrelevant anatomical structures (Fig. 1). This approach minimized background noise and ensured that the CNN models focused on clinically relevant features. The annotated ROI images were then used for model training on two platforms: in Roboflow with the ‘Accurate’ configuration (based on the COCO-S instance segmentation framework) and in Ultralytics with multiple YOLOv11 variants (n, s, m, l, x).

### CNN for mesiodens detection

Convolutional Neural Networks (CNNs) are among the most widely used deep learning architectures in image analysis, particularly due to their outstanding capabilities in visual recognition tasks [1, 17]. Their hierarchical learning structure allows them to automatically extract relevant features from image data, making them highly effective for use in diagnostic imaging applications, including the detection of dental anomalies [16]. 

The object detection model primarily employed in this study was YOLOv11, a state-of-the-art deep learning approach known for its excellent efficiency and accuracy [22]. This model was trained on two different cloud-based environments to provide a comprehensive assessment of its performance in a wide range of scenarios:

- Roboflow: Cloud-hosted service for automating the processing of images as well as the labeling and training of models. The YOLOv11 model was trained with the “Accurate” setup, focusing on high precision [23].
- Ultralytics: A tailored system for training YOLO models that offers advanced settings. The model was implemented with default settings from YOLOv11, which is configured to maximize speed while maintaining accuracy [21].

As seen in other medical image analysis works, the selected YOLO models are meant to provide a trade-off between model complexity and high classification accuracy [16, 24]. The models were trained on datasets that were split into training (70%), validation (20%), and test (10%) groups, and thus, models for this study underwent robust evaluation.

The model’s diagnostic performance was assessed using a confusion matrix, which is a more detailed look at predictions made by the model. This matrix classifies model predictions into four discrete events:

- True Positive (TP): Cases in which the model correctly recognized the presence of mesiodens.
- False Positive (FP): The cases where the model has falsely identified mesiodens in an image annotated as “No Mesiodens.”
- True Negative (TN): Cases correctly identified as “No Mesiodens " by the model.
- False Negative (FN): Cases in which the model did not detect a mesiodens in an image labeled as Mesiodens.

These four categories are fundamental in understanding the diagnostic accuracy of the model and are also used to calculate the performance matrix, including sensitivity (recall), specificity, precision, accuracy, and F1-score.

### Model evaluation metrics

**Fig. 2:**
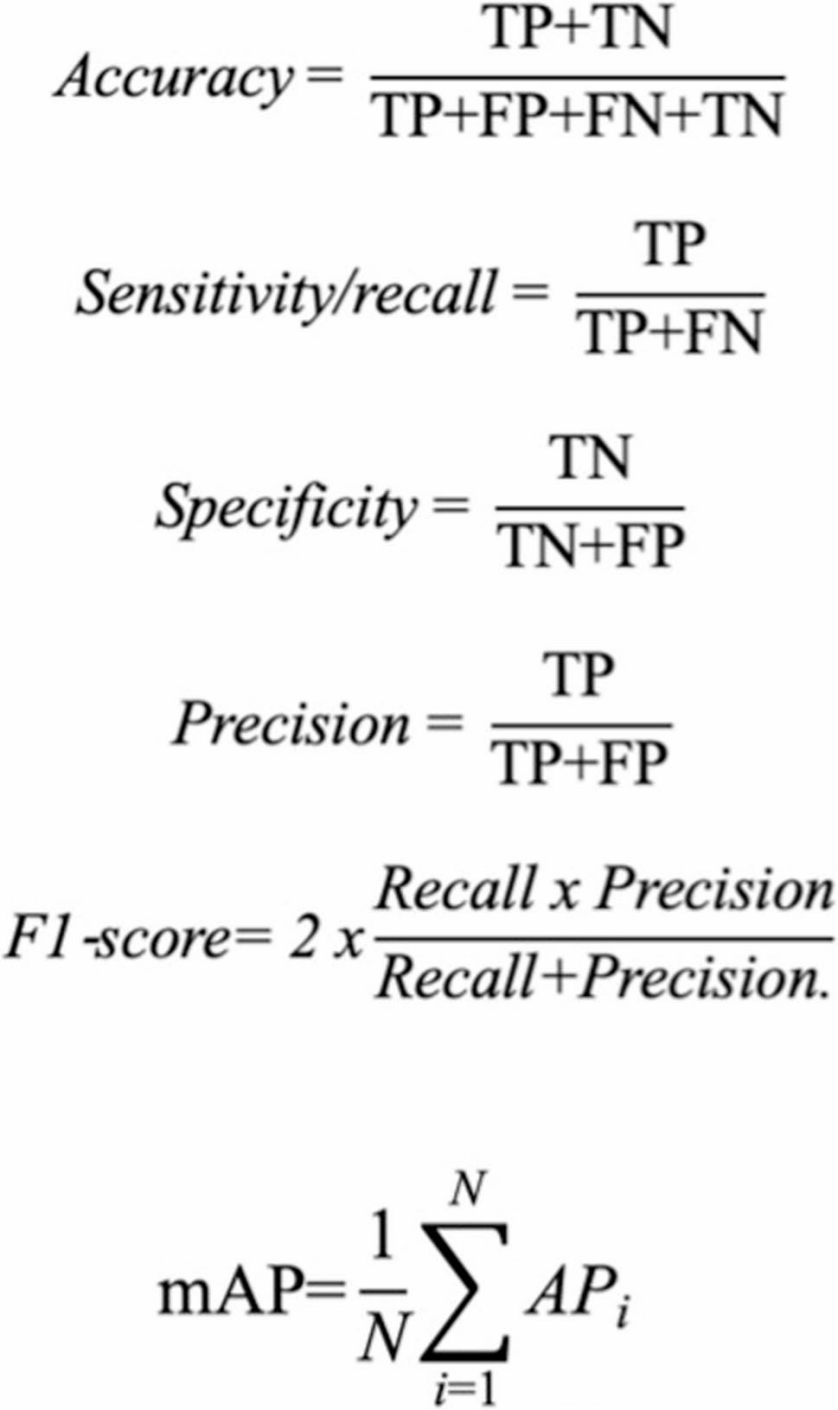
Standard deep learning metric performance formula

Model performance in this study was evaluated using several standard metrics to ensure a comprehensive assessment (Fig. 2):

- Mean Average Precision (mAP) measures the general object detection performance of the model in all classes.
- Precision: the ratio of true positive detections over all the predicted positives, demonstrating the model’s trustworthiness. It’s defined as true positives (TP) divided by the sum of true positives and false positives. Precision showed the model’s reliability in correctly detecting mesiodens.
- Recall (Sensitivity): measures the percentage of actual positives the model correctly identified. Recall represents the model’s ability to identify all the actual instances of mesiodens.
- F1-Score: balances precision and recall for model evaluation.

Confusion matrix: a detailed tabular representation that enumerates the counts of true positives, false positives, true negatives, and false negatives, thereby offering valuable insight into the predictive accuracy of a model [1, 14, 17]. Mean Average Precision (mAP) is a performance metric widely used in object detection tasks. Mean average precision (mAP50) measures the model’s capability with at least 50% overlap between the predicted and ground truth bounding boxes. It quantifies the model’s ability to correctly identify object instances through precision-recall analysis. To compute mAP, predictions are sorted by confidence score, evaluated against ground truth using Intersection over Union (IoU), and the area under the precision-recall curve is calculated. A threshold (e.g., IoU ≥ 0.5) is typically set, and the resulting average precision (AP) is computed for each class. The mAP score is then obtained by averaging the AP values across all classes [1, 25]. 

A comparison of the limitations and advantages of each model was conducted by evaluating the model based on Roboflow and the YOLOv11 models trained in Ultralytics. YOLO model variants were assessed, and performance metrics such as mAP, precision, recall, and F1-score were analyzed. This study aimed to establish the best network architecture and parameters for mesiodens detection, which contributes to selecting the best deep learning paradigm for this task [14]. 

The YOLOv11 family provides models of varying scales (n, s, m, l, x), which differ in network depth, width, and number of parameters. Smaller models (n, s) are optimized for speed and lower computational demand, whereas larger models (l, x) offer improved feature extraction at the cost of higher complexity. Including all five variants allowed us to systematically evaluate the trade-off between computational efficiency and detection performance.

## Result

**Fig. 3:**
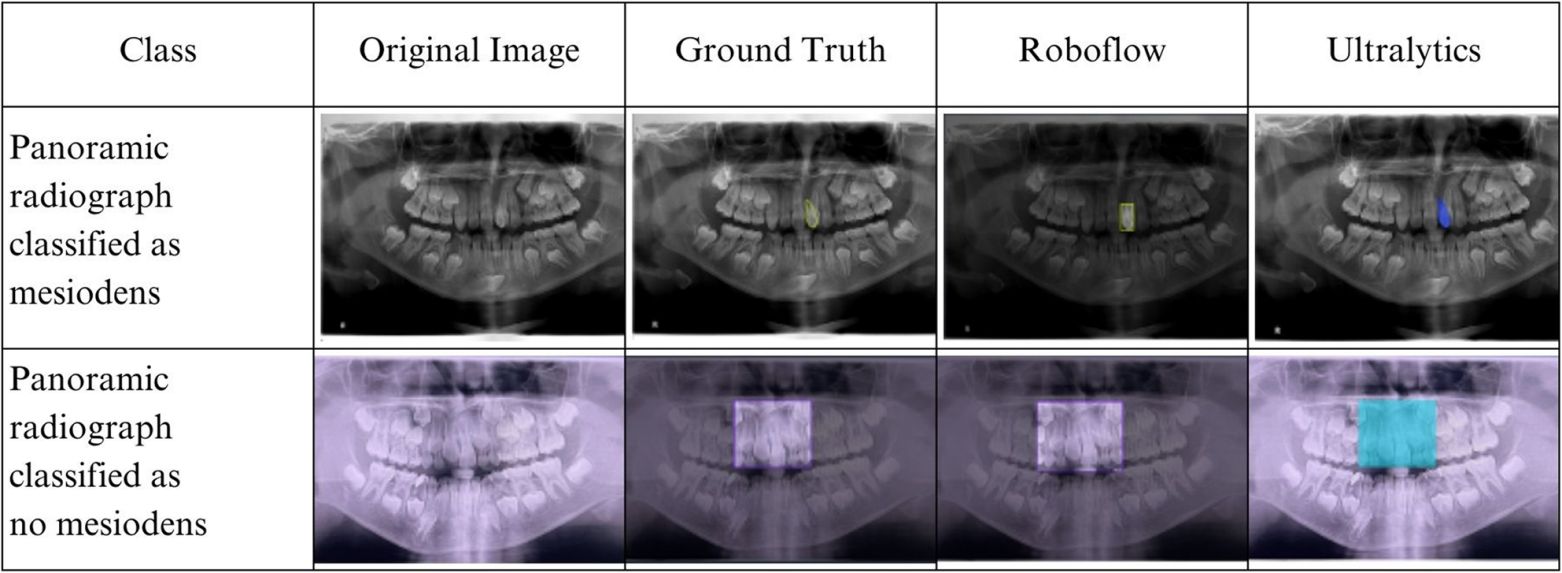
Mesiodens detection on panoramic radiograph using roboflow and ultralytics

In this research, we compare the performance of multiple Convolutional Neural Network (CNN) architectures for mesiodens detection in panoramic radiographs, including Roboflow Accurate, Roboflow YOLOv11 Accurate, and some YOLOv11 variants in the Ultralytics platform (YOLOv11n, YOLOv11s, YOLOv11m, YOLOv11l, and YOLOv11x). Model performance was evaluated on two datasets: the validation set and the inference set. The validation set was used to assess model performance during training, while the inference set simulated real-world clinical conditions to test model generalizability. The prediction outputs from Roboflow and Ultralytics models are illustrated in Fig. 3.

### Model performance evaluation on validation data

**Fig. 4:**
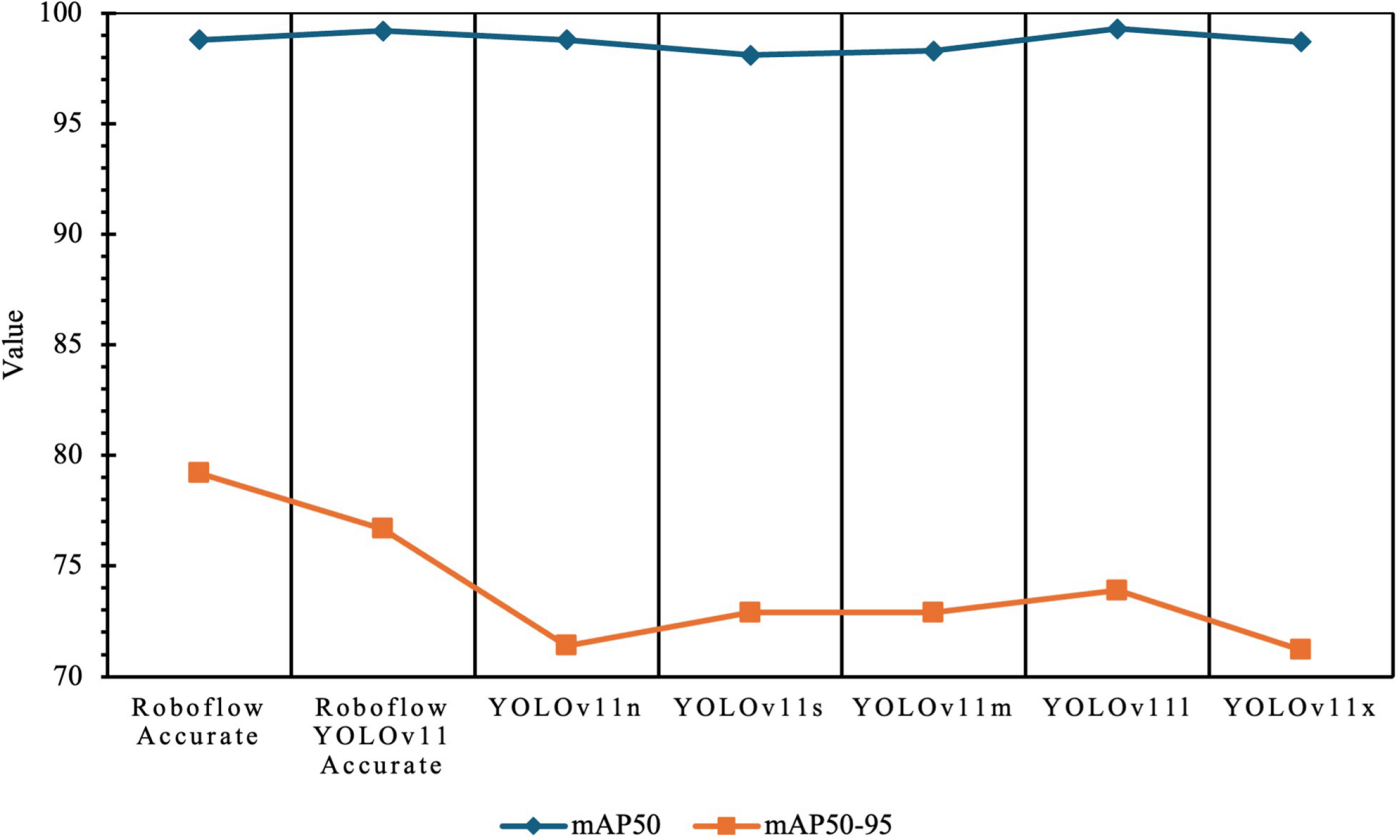
Value of mAP50 and mAP50-95 CNN models

On the validation set, YOLOv11 Accurate achieved the highest performance, with mAP50 of 99.2%, recall of 100%, F1-score of 98.5%, and mAP50–95 of 76.7% (Table 1; Fig. 4). Roboflow Accurate showed comparable results, with mAP50 of 98.8%, recall of 99.1%, F1-score of 98.0%, and a slightly higher mAP50–95 of 79.2%. Ultralytics YOLOv11l reached the highest overall mAP50 at 99.3% but had a lower mAP50–95 of 73.9%. Other YOLOv11 variants (n, s, x) also achieved mAP50 values above 98% but showed lower mAP50–95 scores (71–73%). Collectively, these results indicate that while all models detected mesiodens accurately, their localization precision decreased at higher overlap thresholds.

**Table 1:** Performance of CNN models on validation data

Model	Precision (%)	Recall (%)	F1-Score (%)
Roboflow Accurate	96,90	99,10	97,99
YOLOv11 Accurate	97,00	100,00	98,48
YOLOv11n	95,60	98,20	96,88
YOLOv11s	93,70	97,20	95,42
YOLOv11m	93,30	97,50	95,35
YOLOv11l	97,60	99,10	98,34
YOLOv11x	95,10	94,80	94,95

### Model inference performance evaluation

On the inference set, which simulated real-world application, Ultralytics YOLOv11l demonstrated the most stable performance, with the highest F1-score at 96.8% (Table 2). In contrast, Roboflow YOLOv11 Accurate, despite leading in validation, declined to an F1-score of 86.1%. Roboflow Accurate also showed reduced inference results, with recall of 73.9% and F1-score of 84.3%. Other YOLOv11 variants achieved F1-scores in the range of 91–95%, reflecting consistent detection but slightly less balanced precision–recall compared with YOLOv11l.

**Table 2:** Performance of CNN models on inference data

Model	Precision (%)	Recall (%)	F1-Score (%)
Roboflow Accurate	98.25%	73.91%	84.30%
YOLOv11 Accurate	92.10%	80.85%	86.11%
YOLOv11n	95.08%	97.07%	95.97%
YOLOv11s	97.15%	93.31%	95.15%
YOLOv11m	99.11%	91.91%	95.37%
YOLOv11l	99.11%	94.57%	96.78%
YOLOv11x	98.21%	92.96%	95.51%

## Discussion

When comparing validation and inference outcomes, we found that YOLOv11l demonstrated the most stable performance across both datasets. Despite this YOLOv11 Accurate achieved the highest metrics on validation, its performance dropped considerably on inference, suggesting possible overfitting. Roboflow Accurate also showed satisfactory validation results but underperformed in real-world testing. Taken together, these findings suggest that YOLOv11l provided the most reliable balance between detection performance and generalizability, making it the most promising candidate for clinical application.

Our results showed that while the smaller YOLOv11n and YOLOv11s achieved high mAP50 values, their localization precision (mAP50–95) was lower compared to larger models. This is consistent with prior benchmarks of YOLO architectures, which have reported that lightweight models tend to sacrifice localization precision for speed [21, 22]. YOLOv11x, despite being the most complex, did not surpass YOLOv11l in inference F1-score, suggesting diminishing returns with increasing model size, as also noted in recent comparative analyses of YOLO variants [22]. This highlights the practical advantage of YOLOv11l, which provided the best balance between detection performance and generalizability.

The outcomes of this research support the efficacy of deep learning models in mesiodens detection using panoramic radiographs. Similar to our findings, Ha et al. emphasized the limitations in mesiodens detection and localization, stressing the need for robustness in clinical practice [16, 17]. In our project, two widely used deep learning platforms—Roboflow and Ultralytics—were employed. Roboflow, established in 2020, offers comprehensive tools for image annotation, augmentation, and model training, making it accessible to researchers in medical imaging. Ultralytics, founded in 2018, has become recognized for continuous improvements in the YOLO family of models, the most recent being YOLOv11 (September 2024), and is now one of the most widely used frameworks for object detection [23, 26]. While the dataset split, class structure, image resolution, epoch number, and core augmentations were standardized across both platforms, certain elements of the Roboflow and Ultralytics training pipelines cannot be fully harmonized. To ensure identical data handling, all annotations were exported using Roboflow’s official Ultralytics-compatible YOLO format, providing consistent annotation geometry and class labeling across platforms. As a result, the performance differences observed in this study reflect the combined influence of architecture design and platform-specific training behavior. This comparison therefore represents a practical, real-world evaluation, and future work using fully self-hosted training environments would be valuable to isolate architecture-level effects.

The superior inference performance of YOLOv11l can be attributed to its balanced architecture, which allows a trade-off between detection precision and generalization. Larger models such as YOLOv11l tend to generalize better across diverse data, a feature essential for clinical use [1, 17]. However, the decline in performance of YOLOv11 Accurate and Roboflow Accurate on inference data suggests possible overfitting, where models excel on training features but struggle with unseen cases. Overfitting is a common issue in deep learning, particularly when datasets lack diversity or when models are overly complex [22]. 

Our mAP-based evaluation supports these observations. Despite the fact that YOLOv11 Accurate achieved the highest mAP50, its mAP50–95 was considerably lower (76.7%), indicating reduced localization precision. This is consistent with prior studies highlighting variability in the shape, size, and position of mesiodens, which complicates accurate localization [7, 18, 27]. Roboflow Accurate showed a similar pattern—good validation results but weaker inference performance—aligning with Jeon et al., who noted that strong validation scores do not always translate into robust performance in real-world scenarios due to dataset selection effects [16]. 

Other studies also confirm our results. Ahn et al. reported high performance for automated mesiodens classification but noted that outcomes depended on dataset quality and diversity, similar to our findings [20]. These results underscore the importance of training with diverse, high-quality radiographs and applying advanced augmentation strategies [4, 20, 28]. In our study, Ultralytics YOLOv11l benefitted from its balanced architecture and broader data exposure, yielding the most robust inference performance.

The findings of this study hold significant implications for the clinical diagnosis of mesiodens. Early detection and prompt identification of mesiodens are crucial in preventing complications such as the impaction of adjacent permanent teeth, root resorption, and midline diastema [3, 9]. While the model demonstrated strong performance, achieving high sensitivity, it exhibited varying results on other inference data, indicating the need for further adjustments. Implementing data augmentation, enhancing the model’s ability to handle diverse training data, and utilizing ensemble learning methods may improve detection accuracy [19]. 

However, this study focused on intra-family comparisons among YOLOv11 variants and the Roboflow Accurate model. Two-stage detectors, such as Faster R-CNN and transformer-based architectures, including Detection Transformer (DETR), represent important alternative paradigms with potential advantages in complex medical imaging tasks [29]. The absence of cross-architecture benchmarking is therefore a limitation. Future research should directly compare YOLOv11 with representative two-stage and transformer-based detectors under matched training conditions to more comprehensively evaluate architectural trade-offs and their implications for mesiodens detection.

Despite these promising results, several limitations should be acknowledged. Variability in radiograph quality, contrast, and imaging equipment may influence detection consistency across clinical settings. Differences between the Roboflow and Ultralytics platforms, including preprocessing pipelines and training configurations, may also have contributed to performance variations. The anatomically defined ‘No Mesiodens’ bounding box restricted detection to the conventional anterior region, which means rare ectopic mesiodens located outside this area would not be identified. Detailed exposure parameters such as kVp, mA, and acquisition time were only partially available across the four contributing institutions, introducing potential variability in image characteristics. The dataset was multicenter, yet the number of available images remained limited, indicating the need for larger cohorts and the inclusion of additional centers to enhance model robustness. External validation from independent institutions was not available, and reliance on a single hold-out inference set provides only an initial measure of out-of-sample performance. Future research should incorporate broader multicenter datasets, fully documented imaging parameters, and potentially unconstrained full-arch or multi-stage detection pipelines to strengthen generalizability and clinical applicability.

## Conclusion

This study compared YOLOv11 variants trained on Ultralytics with Roboflow Accurate for automated mesiodens detection in pediatric panoramic radiographs. Ultralytics YOLOv11l demonstrated the best balance of performance and generalizability, achieving the highest inference F1-score, while Roboflow YOLOv11 Accurate showed strong validation results but signs of overfitting on inference data. A key limitation across all models was reduced localization precision at higher overlap thresholds, underscoring the need for further optimization. Future research should expand dataset diversity, integrate advanced architectures such as transformer-based models, and explore ensemble learning strategies to improve robustness and clinical applicability. Additionally, external multicenter validation and diagnostic comparisons with clinicians from different institutions are needed to further confirm the model’s real-world reliability.

## Data Availability

The data supporting the conclusions of this study can be obtained from the corresponding author upon a justified request.

## Abbreviations

AI: Artificial intelligence
CBCT: Cone-beam computed tomography
CNN: Convolutional neural network
COCO: Common objects in context
DETR: Detection transformer
IoU: Intersection over union
mAP: Mean average precision
ROI: Region of interest
SNT: Supernumerary teeth
YOLO: You only look once
